# Disease surveillance using a hidden Markov model

**DOI:** 10.1186/1472-6947-9-39

**Published:** 2009-08-10

**Authors:** Rochelle E Watkins, Serryn Eagleson, Bert Veenendaal, Graeme Wright, Aileen J Plant

**Affiliations:** 1 Curtin Health Innovation Research Institute, Curtin University of Technology, Perth, Australia; 2 Department of Spatial Sciences, Curtin University of Technology, Perth, Australia

## Abstract

**Background:**

Routine surveillance of disease notification data can enable the early detection of localised disease outbreaks. Although hidden Markov models (HMMs) have been recognised as an appropriate method to model disease surveillance data, they have been rarely applied in public health practice. We aimed to develop and evaluate a simple flexible HMM for disease surveillance which is suitable for use with sparse small area count data and requires little baseline data.

**Methods:**

A Bayesian HMM was designed to monitor routinely collected notifiable disease data that are aggregated by residential postcode. Semi-synthetic data were used to evaluate the algorithm and compare outbreak detection performance with the established Early Aberration Reporting System (EARS) algorithms and a negative binomial cusum.

**Results:**

Algorithm performance varied according to the desired false alarm rate for surveillance. At false alarm rates around 0.05, the cusum-based algorithms provided the best overall outbreak detection performance, having similar sensitivity to the HMMs and a shorter average time to detection. At false alarm rates around 0.01, the HMM algorithms provided the best overall outbreak detection performance, having higher sensitivity than the cusum-based Methods and a generally shorter time to detection for larger outbreaks. Overall, the 14-day HMM had a significantly greater area under the receiver operator characteristic curve than the EARS C3 and 7-day negative binomial cusum algorithms.

**Conclusion:**

Our findings suggest that the HMM provides an effective method for the surveillance of sparse small area notifiable disease data at low false alarm rates. Further investigations are required to evaluation algorithm performance across other diseases and surveillance contexts.

## Background

The potential benefits of applying automated monitoring methods to population health data are being increasingly realised, particularly given the growing volume of electronic data that are routinely collected by population health surveillance systems. Models applied to these data need to detect meaningful increases in reported disease incidence quickly using methods which are sensitive to the abnormal aggregation of cases in time and space. Spatio-temporal methods for disease surveillance are thought to offer an improved ability to detect localised events occurring in small regions relative to the temporal surveillance of larger areas [1]. This paper describes the performance of a simple generalised hidden Markov model (HMM) for the surveillance of small area notifiable disease data.

Investigations of spatio-temporal modelling for disease surveillance suggests that simpler approaches which do not require repeated model fitting may be more appropriate for the analysis of large datasets [2]. Spatio-temporal scan statistics which search for evidence of clustering are now commonly used for the detection and evaluation of disease clusters [3]. HMMs also offer a conceptually simple and potentially powerful approach for monitoring sequential data such as those typically generated by surveillance systems. Although HMMs have been proposed for disease surveillance [4,5], they appear to have been rarely used in practice.

HMMs provide a natural way of modelling epidemic and non-epidemic periods by assigning different probability distributions to these two states [4]. The first-order Markovian assumption of HMMs significantly simplifies the time dependency in the data, and the hidden state functions as a switch between the identified states [5]. These models assume that the data are generated from a predetermined number of distributions, and unlike time series approaches, arbitrary choices about the number of transitions between states or their timing are avoided [4]. Importantly for notifiable disease surveillance applications, HMMs are also suitable for applications where the data are sparse.

Good agreement has been found between the performance of HMMs and recognised influenza-like-illness and poliomyelitis epidemics [4]. When compared with methods traditionally used to model the seasonality of influenza outbreaks [6], HMMs which used the hidden variable to eliminate the need for explicit modelling of trend and seasonal effects that can introduce detection bias were found to produce fewer false alarms and be more robust to variations in the data [7]. Rath and coworkers [7] also highlight the advantage of HMMs for automated monitoring over traditional cyclic regression methods in that they can be applied to historical data without the need to distinguish between epidemic and non-epidemic periods.

Bayesian temporal HMMs for disease surveillance have also been proposed [5], illustrating the ease by which these models can incorporate alternative distributional forms. Bayesian methods allow incorporation of prior knowledge and expert opinion, and are most suited to situations where there is some prior knowledge to inform the choice of model parameters. A Bayesian approach may offer advantages in accounting for uncertainty in estimated parameters, as has been recently demonstrated in an application of empirical Bayes methods for the surveillance of multiple data series using statistical process control methods [8]. The approach may also be more robust to changes in the system being monitored over time.

Further research is required to evaluate the use of generalised HMMs for disease surveillance. We aimed to evaluate the performance of a simple Bayesian HMM that requires little baseline data for notifiable infectious disease surveillance.

## Methods

We developed a simple Bayesian HMM for the surveillance of daily reported case counts of hepatitis A in postcode areas in Western Australia. The model was evaluated using simulated outbreaks superimposed on historical baseline data, and outbreak detection performance was compared with the established Early Aberration Reporting System (EARS) algorithms C1, C2 and C3, and a negative binomial cusum. The HMM design, evaluation scenario, comparison algorithms and performance indicators are described in the following sections. This research was approved by the Human Research Ethics Committee of Curtin University of Technology.

### Hidden Markov model

The HMM is based on an existing temporal HMM [9] (pp. 313) which was modified to incorporate spatially referenced data, and is fully described in Additional file 1. As the disease notification data were spatially referenced according to postcode of residence, the spatial structure of the data was incorporated in the model by summing reported cases for each postcode area and any cases reported among its nearest neighbours. Postcode areas which share a common boundary were considered to be nearest neighbours. This process effectively increases the weighting for cases which occur in neighbouring areas in the model.

The HMM requires a valid range of neighbours to be identified for each postcode area in Western Australia. The five postcode areas in Western Australia representing neighbourless islands (Rottnest, Barrow, Thevenard, Cockatoo and Koolan Islands) were allocated as neighbours of the nearest mainland postcode area, representing the usual routes of human movement between these areas. These islands have small or nonexistent resident populations, being predominantly tourism or mining areas, and their treatment has little influence on the results of the analysis.

For simplicity we have restricted the model to a first order HMM where the unobserved disease process is represented by one of two states: an endemic (non-outbreak) state, and an epidemic (outbreak) state. Previous research provides both epidemiological and empirical support for the development of two-state models [4]. We model the distribution of x [t, i] being the sum of observed disease counts in each small area y [t, i] and in area neighbours yn [t, i] at each time point (day) t = 1, ..., t, in each small area i = 1, ..., n, in the two disease states as Poisson. The Poisson model is commonly used for count data, however this model implies that the variance of x [t, i] is equal to its expected mean. Descriptive analysis of the historical hepatitis A notification data suggest that they are over-dispersed, and fluctuating variance has been reported as problematic for automated disease surveillance [10]. A gamma prior distribution for the means of each state in the model was used to describe extra variation in the data that can not be explained by the Poisson assumption.

Using a Bayesian approach requires specification of prior distributions for unknown model parameters, which include the mean of each of the two hidden disease states, and the transition matrix which governs movement between the two hidden states. Prior distributions reflect our uncertainty about the unknown parameter values, and can be used to include expert knowledge or parameters derived from historical data in the analysis. We evaluated the models using relatively uninformative priors which were designed to provide limited information about the value of the unknown parameters.

Movement between the two disease states in the model is governed by a stationary transition matrix which determines the transition probabilities. An uninformed Dirichlet prior distribution was used for the prior on the initial transition probabilities, which is a generalisation of the beta distribution to K variables, with each assuming a value between 0 and 1. For the priors on the subsequent transition probabilities we used a gamma equivalent to the Dirichlet [9].

Gamma priors on the means for the baseline and outbreak states were selected to describe a small probability of more extreme state mean values, however other distributions could be used. The relatively uninformative priors gamma(10,10) and gamma(40,20) were used, which produced distribution means of 1 and 2 respectively, with a common variance of 0.1. Due to the sparse nature of the baseline dataset, the prior means for the baseline and outbreak states were approximately 5 and 10 times greater than the baseline mean respectively. The prior mean for the outbreak state was also equal to the maximum daily case count of the baseline dataset. To investigate the influence of these parameters on the model performance, the HMM was also tested using a prior mean of 3 for the outbreak state in the 7 and 14-day models, and a prior mean of 4 for the outbreak state in the 28-day baseline model. Due to the potential problem of label switching, the prior mean for the outbreak disease state was constrained to be greater than the mean for the baseline disease state, as has been used previously [5,9]. The model was implemented in WinBUGS 1.4.3 [11] (available from http://www.mrc-bsu.cam.ac.uk/bugs/winbugs/contents.shtml).

We aimed to develop a simple maintenance-free HMM that has general application and requires little baseline data. As such, no covariates were included in the model. Like other algorithms [3], the model has been simplified by assuming that the population at risk remains stable over time. However, these models can be easily extended to include covariates such as population denominators, or day-of week effects.

To limit the computing resources required for analysis, the HMM is evaluated here using just 7 temporal data points during any single analysis. This is implemented as a moving 7-day analysis window which progresses from analysing days 1–7 through to analysing days 144–150 of the test datasets. This provides baseline data requirements for the HMM that are comparable with the EARS cusums. We also evaluate the use of additional baseline data in the model by aggregating 14 days of baseline data into seven consecutive 2-day units, and 28 days of baseline data into seven consecutive 4-day units, to allow efficient analysis using the 7-day model structure.

The Markov Chain Monte Carlo (MCMC) sampling algorithm was run for an initial 1000 iterations before model parameters were monitored for a further 1000 iterations. The results based on this sample from the posterior distribution were used for model inference. Tests of the model using two chains with different initial values suggested that the chains quickly converged, and the sampling was sufficient to produce stable results for trial purposes while minimising the time required to run the model. The time required for one analysis of daily data from the 383 areas using a Pentium 4 3.0 GHz computer with 1 gigabyte of RAM was approximately 2 minutes. We used the R statistical analysis software version 2.7.1 [12] and the R2WinBUGs package [13] to automate prospective analysis of the test datasets.

### Evaluation scenario

The model was evaluated using semi-synthetic data. Simulated outbreaks of hepatitis A were superimposed on historical baseline data for hepatitis A in Western Australia extracted from the National Notifiable Diseases Surveillance System. National notifiable disease surveillance data in Australia is spatially referenced by residential postcode to protect individual confidentiality. There were no reported outbreaks in Western Australia during the 150-day baseline period (January to May 2004); however, the presence of small undetected outbreaks cannot be excluded.

A total of 31 cases of hepatitis A were notified during the baseline period, producing an average notification rate of 0.2 cases per day, and a maximum of two cases were notified on any single day during the baseline period. The HMM analysed the semi-synthetic data as if the data were being received prospectively by date of report.

Historical notification data are limited in that notification of cases is dependent upon a number of processes which are required before a person who becomes ill seeks help, receives an accurate diagnosis and is notified to health authorities. Notification data are also not directly related to the population at risk, as they depend on health service use and provision, and reflect the use of targeted screening and contact tracing activities.

A spatial stochastic simulation model programmed in MapBasic version 8.0 (MapInfo Corporation, 2005) was used to simulate 4 different outbreak scenarios for performance evaluation. Outbreaks of hepatitis A were simulated using a state-transition model based on household address point data for Western Australia to ensure a realistic distribution of cases in space [14]. The model was parameterised based on a mean incubation period of 28 days, with a range from 15 to 50 days, consistent with documented parameters [15]. A latent period of between 7 and 29 days (with a most likely value of 14 days) and an infectious period of between 14 and 21 days (with a most likely value of 17 days) were parameterised using beta-PERT distributions [16] (pp. 275). Transition to the symptomatic state was parameterised based on the onset of infectiousness to preserve the known association between decreasing infectiousness and the commencement of symptoms [15]. Infectious cases had an average of 5 close contacts per day which was Poisson-distributed. The detailed process of case detection was not modelled, and all outbreak cases were assumed to be detected on the day that they became symptomatic. For each simulation, the index case was randomly allocated to a spatial location based on the distribution of all households in Western Australia.

To allow analysis of the influence of outbreak characteristics on algorithm performance, simulation parameters were varied to create four outbreak scenarios based on two factors: the degree of clustering of cases, and the size of the outbreak. The four outbreak scenarios compared were labelled according to the characteristics of the simulated outbreaks: small less clustered (S1), small more clustered (S2), large less clustered (S3) and large more clustered (S4). Population susceptibility to infection was specified as 5 per cent for the smaller outbreak scenarios and 10 per cent for the larger outbreak scenarios. The geographic area within which an infectious individual could infect susceptible individuals was specified as a circular buffer with its radius randomly varying between 5 and 10 km for more clustered outbreaks, and 10 and 20 km for less clustered outbreaks.

One hundred outbreaks were simulated for each outbreak scenario, with the index case randomly inserted between day 35 and 85, so that the earliest possible notification of the first case would occur on day 50 of the historical baseline. From 35 days following infection of the index case, outbreak control measures were introduced to eliminate the outbreak and limit the proportion of the test datasets that represented outbreak days.

**Figure 1:**
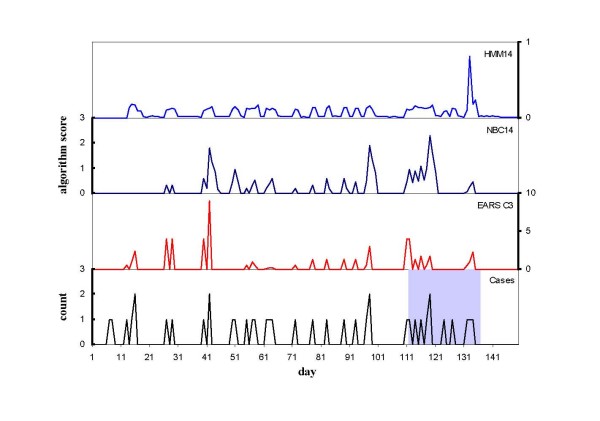
**Hepatitis A case notifications, outbreak period (shaded) and scores for the EARS C3, 14-day negative binomial and 14-day hidden Markov model algorithms for simulation 15 from the smaller less clustered simulation scenario (S1)**.

**Figure 2:**
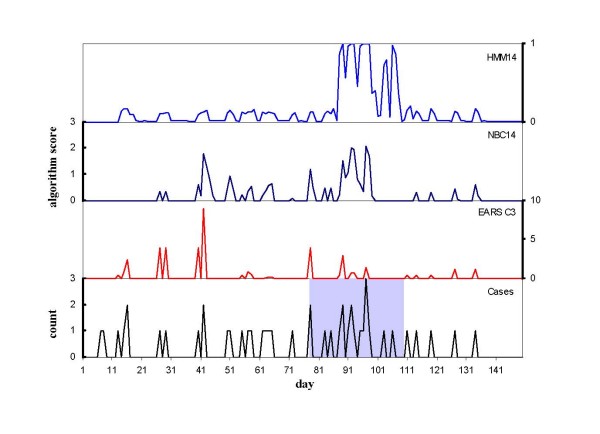
**Hepatitis A case notifications, outbreak period (shaded) and scores for the EARS C3, 14-day negative binomial and 14-day hidden Markov model algorithms for simulation 78 from the larger more clustered simulation scenario (S4)**.

From among the 100 simulated datasets for each outbreak scenario, datasets with 5 or more outbreak cases were selected for analysis to provide a standard minimum outbreak size for evaluation which is of particular epidemiological interest due to the potential for early detection and disease control interventions. The number of simulated outbreaks used to evaluate the algorithms for the four scenarios was then 64, 62, 95 and 93 for the small less clustered, small more clustered, large less clustered and large more clustered scenarios respectively. The temporal profile of outbreaks from the smaller and larger outbreak scenarios is illustrated in Figures 1 and 2. Smaller outbreak scenarios had an average of 8.1 cases per outbreak (standard deviation = 2.9, range = 5–16), and larger outbreak scenarios averaged 16 cases per outbreak (standard deviation = 8.0, range 5–39). The average interval between the first two cases that were classified as outbreak cases (cases 2 and 3) was 4.5 days for the smaller scenarios and 3.5 days for the larger scenarios. The average intervals between cases 2 and 4, and 2 and 5 were 8.1 and 11.2 days for the smaller outbreak scenarios, and 6.1 and 8.2 days for the larger outbreak scenarios respectively.

For each evaluation dataset the results of the daily HMM analysis, being the daily probability of each postcode area being in an outbreak state, was monitored. Summary statistics were generated based on the maximum daily HMM value to enable comparison of performance between different scenarios and outbreak detection algorithms.

### Comparison algorithms

The EARS C1, C2 and C3 algorithms [17] were used to provide an established standard with which to compare algorithm performance. The EARS cusums require limited baseline data, and are widely used for monitoring population health surveillance data, including notifiable disease data in New Zealand [18]. The algorithms were run using the R statistical software based on the implementation of the algorithms in the EARS-X Microsoft Excel software version, which can be downloaded from http://www.bt.cdc.gov/surveillance/ears/downloadearsx.asp. A negative binomial cusum (NBC) [19] based on an existing model [20] was also selected for comparison due to the potential ability of this method to minimise false alarms. The R code for the EARS and negative binomial cusums is provided in Additional file 1.

The EARS algorithms were originally designed to signal an aberration when the cusum values exceed 2, which implies that the surveillance data have exceeded a level which is three standard deviations greater than the baseline mean. Although the cut-off value used to determine signalling of the cusums was varied to allow exploration of performance at different false alarm rates, the implementation of the C3 algorithm retains the threshold of 2 used to exclude large observed counts on the current or previous two days from the cusum total score, as implemented in the EARS-X software (see Additional file 1).

The negative binomial cusum traditionally signals when the cumulative sum value exceeds a predetermined threshold which is found by specifying the desired in-control average run length, and monitoring for a change in the over-dispersion parameter 'c' from a specified in control level to a specified out of control level. The negative binomial cusum was calibrated to an out of control state being two standard deviations greater than the historical mean, which was estimated based on the most recent 7, 14 or 28 days of data.

### Performance indicators

Performance was summarised over multiple simulation datasets for each outbreak scenario. Averaging outbreak detection performance over the datasets with randomly inserted outbreaks eliminates bias due to the timing and location of the outbreak in the baseline dataset, and allows estimation of the average expected detection performance of the models using different alarm thresholds given the baseline data. The results from these trials were used to describe model performance and the relationship between sensitivity, timeliness and false alarms.

Performance comparisons were based on two main indicators: sensitivity, which describes the ability of the algorithm to detect simulated outbreaks at any time during each outbreak period; and timeliness, which describes the number of days from the beginning of each outbreak period until the first signal for each outbreak. As timeliness was only able to be calculated for outbreaks that were detected, an additional outcome variable 'adjusted timeliness' was derived to enable the generation of complete timeliness data by allocating a value of 28 days as the timeliness result if an outbreak was undetected.

The outbreak period was defined as beginning on the day that the second outbreak case was inserted, and ending on the third day following the detection of the last outbreak case. As such, outbreaks occurred on days when epidemiological linkages were present for two or more cases. Any signals that occurred during the outbreak period were considered valid, and the first of these was used to calculate the time to detection. Signals that occurred on non-outbreak days were considered false alarms, and the average proportion of false alarms was calculated as the total number of false alarms divided by the total number of non-outbreak days for each simulated dataset. To compare algorithm performance, empirical methods were used to determine the signalling threshold cut-off values for each algorithm that would produce equivalent false alarm rates. We investigate the performance of both algorithm types using false alarm rates of approximately 0.05 and 0.01, which implies that threshold values were selected so that the algorithms are expected on average to produce a false positive alarm approximately once every twenty and once every one hundred non-outbreak days respectively.

The performance of each algorithm was also evaluated by comparing the area under the receiver operating characteristic (ROC) curve (AUC) (Table 1) [21]. The AUC calculations were performed using the trapz function of the CaTools package for the R statistical software [22]. To allow the consideration of both sensitivity and timeliness in AUC comparisons, a combined AUC indicator (CAUC) was also generated [21], which weights the contribution of the sensitivity component of the AUC indicator based on the proportion of time saved relative to a reference value. As no historical data were available to provide a reference value, a fixed value of 10 days was used. The weightings applied to the sensitivity data were calculated as (10-timeliness)/10, with a lower limit of zero. As such, outbreaks detected more than ten days after their commencement contribute no value to the CAUC indicator. Both AUC and CAUC indicators were calculated for false alarm rates between 0 and 0.1, representing the most relevant portion of the ROC curve for disease surveillance applications.

**Table 1:** Algorithm area under the ROC curve (AUC) and Combined AUC (CAUC) performance statistics for false alarm rates between 0 and 0.1 by simulation scenarios S1–S4.

	mean (median) AUC_0–0.1 _× 10^-3^				mean (median) CAUC_0–0.1 _× 10^-3^			
Algorithm	S1	S2	S3	S4	S1	S2	S3	S4

EARS C1	0.0 (0.0)	0.0 (0.0)	0.0 (0.0)	0.04 (0.0)	0.0 (0.0)	0.0 (0.0)	0.0 (0.0)	0.0 (0.0)
EARS C2	0.3 (0.0)	0.3 (0.0)	0.3 (0.0)	0.3 (0.0)	0.2 (0.0)	0.2 (0.0)	0.1 (0.0)	0.2 (0.0)
EARS C3	0.3 (0.0)	0.3 (0.0)	0.3 (0.0)	0.4 (0.0)	0.2 (0.0)	0.2 (0.0)	0.1 (0.0)	0.2 (0.0)
NBC 7	3.6 (0.0)	3.4 (0.0)	5.2 (8.1)	5.6 (8.2)	1.5 (0.0)	1.6 (0.0)	1.9 (0.0)	2.1 (0.0)
NBC 14	5.1 (8.1)	4.8 (8.1)	6.0 (8.8)	6.3 (8.7)	2.6 (0.0)	2.5 (0.0)	2.4 (0.0)	2.6 (0.0)
NBC 28	4.7 (4.5)	4.7 (4.5)	2.9 (0.0)	3.1 (0.0)	2.2 (0.0)	2.5 (0.0)	1.4 (0.0)	1.7 (0.0)
HMM 7	6.0 (7.7)	5.8 (7.7)	7.6 (8.3)	7.7 (8.3)	2.3 (0.0)	2.0 (0.0)	3.2 (1.8)	2.9 (1.8)
HMM 14	7.5 (8.3)	8.1 (8.4)	7.9 (8.7)	8.3 (8.8)	3.5 (3.5)	3.9 (4.1)	3.6 (3.4)	4.1 (4.4)
HMM 28	5.2 (8.9)	6.2 (9.1)	4.3 (0.0)	4.9 (8.8)	2.4 (0.0)	3.2 (0.0)	2.1 (0.0)	2.5 (0.0)

ROC: receiver operating characteristic

Simulation scenarios S1:smaller less clustered; S2:smaller more clustered; S3:larger less clustered; S4:larger more clustered.

To limit the number of statistical tests conducted, formal statistical comparisons of AUC, CAUC, sensitivity, and timeliness were performed for six of the algorithms tested: the three HMMs, the best performing EARS algorithm (C3) and two negative binomial cusum algorithms (7 and 14-day models). Comparisons were made using Friedman Rank Sum Tests as implemented in the Stats package for the R software version 2.7.1 [12], as the parametric model requirements of normality cannot be assumed. Selected 2-sided multiple comparisons were performed using paired Wilcoxon Signed Rank tests to investigate difference in performance between the three HMMs and three other algorithms, including comparisons between HMMs of different baseline lengths. As these twelve comparisons were conducted for each analysis, the two-tailed p-value used to determine significance for the Wilcoxon Signed Rank tests was adjusted to reflect the repeated testing and set at 0.05/12, or 0.0042.

## Results

Figures 1 and 2 illustrate the daily signalling pattern of the HMM, NBC and EARS algorithms for two randomly selected simulations for the smaller less clustered and larger more clustered simulation scenarios respectively. Compared with the cusum-based algorithms which respond quickly to changes in the incidence of cases, the HMM responds most strongly to the accumulation of cases in close proximity.

**Figure 3:**
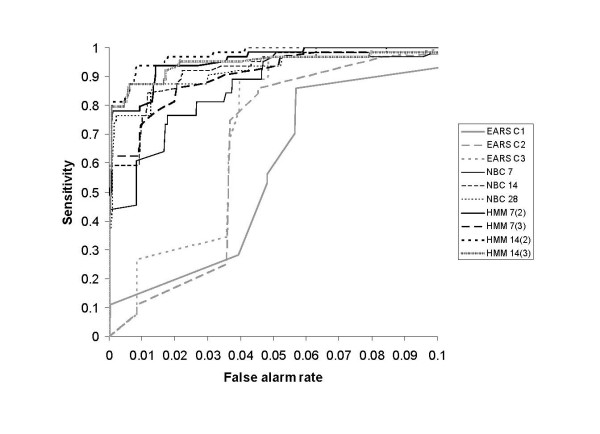
**Sensitivity of the outbreak detection algorithms according to false alarm rates less than 0.1 for the smaller less clustered simulation scenario (S1)**.

**Figure 4:**
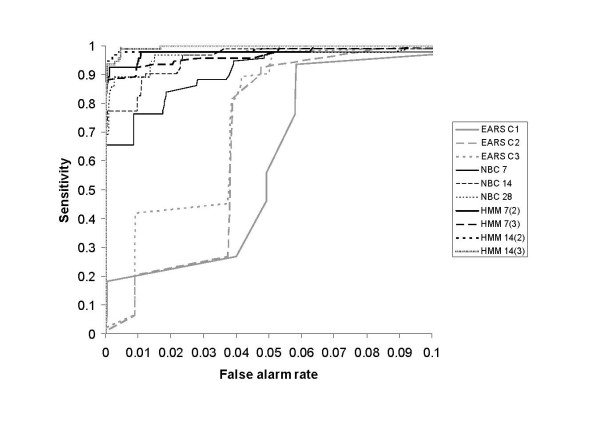
**Sensitivity of the outbreak detection algorithms according to false alarm rates less than 0.1 for the larger more clustered simulation scenario (S4)**.

The sensitivity of the algorithms for false alarm rates less than 0.1 is summarised for the smaller less clustered and larger more clustered simulation scenarios in Figures 3 and 4 respectively. The sensitivity of the 28-day HMM is not displayed in Figures 3 and 4 as it was indistinguishable from that of the 14-day HMM with a prior mean of 3. The shorter-baseline HMMs were more sensitive when the smaller outbreak prior mean was used; however, there was no appreciable difference for both prior means tested for the 28-day HMM. The following results present the performance of the 7, 14 and 28-day HMMs using the outbreak state prior means of 2, 2, and 3 respectively.

### Area under the ROC curve

There was a significant difference in AUC_0–0.1 _among the algorithms formally compared for all simulation scenarios (S1 Friedman χ^2 ^= 120.2, p < 0.00001, S2 Friedman χ^2 ^= 131.8, p < 0.00001, S3 Friedman χ^2 ^= 157.5, p < 0.00001 and S4 Friedman χ^2 ^= 175.8, p < 0.00001). For all simulation scenarios, the 14-day HMM had significantly greater AUC than the 7-day HMM (all p < 0.00001). For both larger outbreak simulation scenarios, the 14-day HMM also had significantly greater AUC_0–0.1 _than the 28-day HMM (both p ≤ 0.0009), and the 7-day HMM had significantly greater AUC_0–0.1 _than the 28-day HMM for the larger less clustered scenario (p = 0.0004) but not the larger more clustered scenario after adjustment for multiple testing (p = 0.005).

All HMMs had a significantly greater AUC_0–0.1 _than the EARS C3 algorithm (all p ≤ 0.00001) for all simulation scenarios. The 14-day HMM also had significantly greater AUC_0–0.1 _than the 7-day NBC (all p ≤ 0.00001) for all simulation scenarios, and the 28-day HMM had a significantly greater AUC_0–0.1 _than the 7-day NBC for both smaller simulation scenarios (both p ≤ 0.004).

### Combined area under the ROC curve

There was a significant difference in CAUC_0–0.1 _among the algorithms compared for all simulation scenarios (S1 Friedman χ^2 ^= 77.3, p < 0.00001, S2 Friedman χ^2 ^= 96.1, p < 0.00001, S3 Friedman χ^2 ^= 109.4, p < 0.00001 and S4 Friedman χ^2 ^= 118.4, p < 0.00001). For all simulation scenarios, the 14-day HMM had significantly greater CAUC_0–0.1 _than the 7-day HMM (all p ≤ 0.0002). For both larger outbreak simulation scenarios, the 14-day HMM also had significantly greater CAUC_0–0.1 _than the 28-day HMM (both p ≤ 0.002).

For all simulation scenarios, all HMMs had a significantly greater CAUC_0–0.1 _than the EARS C3 algorithm (all p ≤ 0.00002). The 14-day HMM also had significantly greater CAUC_0–0.1 _than the 7-day NBC for all simulation scenarios (all p ≤ 0.0002). For both larger outbreak simulation scenarios, the 14-day HMM had significantly greater CAUC_0–0.1 _than the 14-day NBC (both p ≤ 0.001), and the 7-day HMM had significantly greater AUC_0–0.1 _than the 7-day NBC (p ≤ 0.0008).

### Sensitivity and timeliness

The sensitivity and timeliness of the algorithms tested for false alarm rates approximating 0.05 and 0.01 are summarised in Tables 2 and 3, and Additional file 1. Algorithm performance was most strongly dependent on the desired false alarm rate for surveillance, followed by the size of the outbreak and the extent of clustering. As such, performance is described below according to two false alarm rates that are commonly used for performance evaluation.

**Table 2:** Algorithm performance statistics for smaller less clustered outbreaks (S1:n = 64) for false alarm (FA) rates approximating 0.05 and 0.01.

Algorithm	mean FA rate	mean sensitivity	mean (median) timeliness	mean (median) adjusted timeliness
EARS C1	0.048	56.3	6.4 (0)	15.8 (19.5)
EARS C2	0.045	85.9	6.0 (2)	9.1 (6)
EARS C3	0.049	96.9	3.0 (0)	3.8 (0)
NBC 7	0.050	96.9	3.7 (1)	4.5 (1)
NBC 14	0.049	96.9	3.9 (1)	4.6 (1)
NBC 28	0.052	93.8	4.8 (1)	6.3 (1)
HMM 7	0.049	98.4	3.5 (1)	3.9 (1)
HMM 14	0.051	100	5.0 (2)	5.1 (2)
HMM 28	0.050	89.1	6.9 (5)	9.2 (6)

EARS C1	0.0004	10.9	4.4 (3)	25.4 (28)
EARS C2	0.008	10.9	3.0 (2)	25.3 (28)
EARS C3	0.008	26.6	4.3 (2)	21.7 (28)
NBC 7	0.008	60.9	7.7 (4)	15.6 (16.5)
NBC 14	0.010	75.0	5.6 (2.5)	11.2 (7.5)
NBC 28	0.012	76.6	8.2 (6)	12.9 (10)
HMM 7	0.009	79.7	8.5 (7)	12.5 (11)
HMM 14	0.008	93.8	7.3 (5.5)	8.6 (6)
HMM 28	0.010	89.1	7.1 (5)	9.4 (6)

**Table 3:** Algorithm performance statistics for larger more clustered outbreaks (S4:n = 93) for false alarm (FA) rates approximating 0.05 and 0.01.

Algorithm	mean FA rate	mean sensitivity	mean (median) timeliness	mean (median) adjusted timeliness
EARS C1	0.049	55.9	6.2 (2.5)	15.8 (18)
EARS C2	0.048	92.5	5.0 (4)	6.7 (4)
EARS C3	0.050	93.5	2.8 (0)	4.4 (1)
NBC 7	0.050	97.8	2.7 (1)	3.3 (1)
NBC 14	0.050	98.9	2.6 (1)	2.9 (1)
NBC 28	0.055	97.8	3.5 (1)	4.0 (1)
HMM 7	0.049	97.8	4.2 (3)	4.7 (3)
HMM 14	0.049	98.9	4.3 (3)	4.6 (3)
HMM 28	0.049	100	4.7 (3)	4.7 (3)

EARS C1	0.0004	18.3	7.8 (9)	24.3 (28)
EARS C2	0.009	20.4	8.4 (5)	24.0 (28)
EARS C3	0.009	41.9	7.1 (4)	19.2 (28)
NBC 7	0.009	76.3	7.2 (5)	12.1 (10)
NBC 14	0.010	82.8	6.6 (5)	10.3 (7)
NBC 28	0.013	89.2	7.2 (6)	9.4 (7)
HMM 7	0.010	94.6	6.6 (5.5)	7.7 (6)
HMM 14	0.008	97.8	4.9 (4)	5.4 (4)
HMM 28	0.010	100	5.0 (4)	5.0 (4)

#### 0.05 false alarm level

At false alarm rates approximating 0.05, there was little difference in the sensitivity of the algorithms tested, apart from EARS C1, which had consistently lower sensitivity than all other algorithms. Among the algorithms compared, there was a significant difference in sensitivity for the smaller less clustered and larger more clustered simulation scenarios (S1 Friedman χ^2 ^= 15.7, p = 0.008, S2 Friedman χ^2 ^= 8.1, p = 0.15, S3 Friedman χ^2 ^= 8.6, p = 0.13 and S4 Friedman χ^2 ^= 12.7, p = 0.03); however, all multiple comparisons were non-significant following adjustment for multiple testing (all p > 0.008).

There was a significant difference in timeliness among the algorithms compared for all simulation scenarios at the 0.05 false alarm rate (S1 Friedman χ^2 ^= 53.4, p < 0.00001, S2 Friedman χ^2 ^= 60.6, p < 0.00001, S3 Friedman χ^2 ^= 70.1, p < 0.00001 and S4 Friedman χ^2 ^= 76.7, p < 0.00001), with the HMM on average detecting outbreaks several days later than the EARS C3 and NBC algorithms. Multiple comparisons for the both smaller simulation scenarios found the 28-day HMM had a significantly greater time to detection than all other algorithms (all p ≤ 0.002), with the exception of the 7-day HMM (p = 0.22) for the smaller more clustered simulation scenario. The 7-day HMM also had a significantly greater time to detection than the EARS C3 algorithm and the 7 and 14-day NBCs (p < 0.001) for the smaller less clustered simulation scenario.

For the larger simulation scenarios, the 14 and 28-day HMMs had significantly greater time to outbreak detection than the EARS C3 and 7 and 14-day NBCs (p < 0.002). For the larger more clustered scenario the 7-day HMM also had significantly greater time to detection than the EARS C3 and 7 and 14-day NBCs (p < 0.002). For the larger less clustered scenario, the 7-day HMM had a significantly lower time to detection than the 14 and 28-day HMMs (both p < 0.004).

#### 0.01 false alarm level

There was a significant difference in sensitivity among the algorithms compared for all simulation scenarios at the 0.01 false alarm level (S1 Friedman χ^2 ^= 103.4, p < 0.00001, S2 Friedman χ^2 ^= 119.6, p < 0.00001, S3 Friedman χ^2 ^= 140.3, p < 0.00001 and S4 Friedman χ^2 ^= 168.6, p < 0.00001). The smaller more clustered scenario was the only scenario to show a significant difference in performance between the different HMMs, with the sensitivity of the 7-day HMM significantly lower than both the 14 and 28-day HMMs (both p ≤ 0.001).

For all simulation scenarios, all HMMs had significantly greater sensitivity than the EARS C3 algorithm (all p ≤ 0.00001), and the 14-day and 28-day HMMs had significantly greater sensitivity than the 7-day NBC (all p ≤ 0.00001). For both the larger simulation scenarios, the 7-day HMM also had significantly greater sensitivity than the 7-day NBC (both p ≤ 0.00001). For all simulation scenarios except the smaller less clustered scenario, the 14 and 28-day HMMs also had significantly greater sensitivity than the 14-day NBC (all p ≤ 0.00001).

At the 0.01 false alarm level, there was a significant difference in timeliness among the algorithms compared for the larger simulation scenarios only (S1 Friedman χ^2 ^= 5.1, p < 0.41, S2 Friedman χ^2 ^= 9.3, p < 0.10, S3 Friedman χ^2 ^= 20.3, p < 0.001 and S4 Friedman χ^2 ^= 17.1, p < 0.004). For the larger simulation scenarios the 14 and 28-day HMMs had a significantly lower time to detection than the 7-day HMM (both p < 0.002), and the 14-day HMM also had a significantly lower time to detection than both the 7 and 14-day NBCs (all p < 0.0042). For the larger less clustered scenario the 28-day HMM also had a significantly lower time to detection than the 7-day NBC (p = 0.0008), and for the larger more clustered scenario the 28-day HMM also had a significantly lower time to detection than both the 7 and 14-day NBCs (both p < 0.003).

## Discussion

The proposed HMM which uses Bayesian methods to estimate the model parameters provides a conceptually simple basis for the surveillance of small area disease notification data. Unlike the cusum-based comparison models, HMMs can benefit from the spatial information associated with the postcode-based disease notification data. Overall performance comparisons based on the AUC and CAUC indicators at false alarm rates less than 0.1 found the 14-day HMM provided the best outbreak detection performance, with the area under the receiver operator characteristic curve being significantly greater than the EARS C3 and 7-day negative binomial cusum algorithms for all AUC and CAUC comparisons.

The desired false alarm rate for surveillance was the most important determinant of the best performing algorithm. The specific outbreak scenario was still an important source of performance differences, with the size of the outbreak having a greater impact on performance differences between algorithms than the extent of clustering. At higher false alarm rates, the cusum-based algorithms provided significantly earlier outbreak detection compared with the HMMs. At lower false alarm rates the HMMs provided significantly higher sensitivity across all simulation scenarios compared with the EARS C3 and negative binomial cusums, and significantly earlier outbreak detection among the larger outbreak scenarios compared with the negative binomial cusum.

Our findings are similar to previous comparative studies which suggest that the EARS algorithms detect events quickly and have a relatively high rate of false alarms [23,24]. The consistent and early detection of outbreaks at low false alarm rates remains challenging. A large-scale simulation study found that the algorithms tested did not reliably detect outbreaks of interest across a wide range of scenarios at low alert rates [25], and a similar decline in algorithm performance at low false alarm rates, particularly among the EARS algorithms, was observed in this study.

The use of longer series of baseline data for automated surveillance is recognised to provide less volatile baseline estimates [26], and a review suggests that few methods demonstrate reliable detection using short-term baseline data [27]. Although the HMMs that analysed more than 7 days of data were found to have better overall performance than the 7-day HMM, model performance was not significantly improved by analysing more than 14 days of data, possibly due to the effect of data aggregation. The 14-day negative binomial cusum also provided improved performance over the 7-day negative binomial cusum, and other investigations with cusum-based models have demonstrated that performance quickly improves as the baseline estimation period is increased to more than 7-days [23].

The scope for improving the performance of the EARS algorithms by increasing the amount of historical data used remains to be investigated; however, our results demonstrate that at low false alarm rates, equivalent short baseline models for both the HMM and the NBC demonstrate significantly improved performance compared with the EARS algorithms, indicating that the limited baseline period alone is insufficient to explain the performance differences found. Comparative analysis of algorithm performance using common datasets is an important means of identifying the distinguishing performance characteristics of different algorithms and enabling the selection of complementary algorithms in surveillance systems. The absence of standard evaluation scenarios limits wider comparison of our study findings, and further performance comparisons with alternative HMMs and spatio-temporal methods such as SaTScan [3] are required. Additional work is needed to investigate the potential benefits of using a HMM structure of longer than 7 days, and the factors which determine the optimal baseline period for monitoring; including baseline characteristics such as periodicity, and disease-specific factors such as generation time.

Applications of the HMM require prior means to be specified for the outbreak and non-outbreak states of the HMM. Our findings suggest that HMM performance was generally better when lower prior means were specified; however, longer baseline models were less sensitive to changes in the prior mean, with the 28-day HMM demonstrating little difference in performance between the two prior means trialled. Although we attempted to compare algorithms across a variety of outbreak scenarios which were based on known disease transmission parameters and would not favour any single algorithm, we cannot rule out the use of scenarios which may have favoured the HMM. Further algorithm comparison is required with respect to different outbreak data, diseases and spatial contexts before more general performance descriptions can be made. The current study is limited in the use of only a single typical series of baseline data in the evaluations, and the study of a single disease. The performance evaluation process also excluded very small outbreaks of less than 5 cases, and although this could affect algorithm comparison results, this would be limited to events of lesser public health importance.

Evaluation using epidemiologically and spatially appropriate data is important to ensure that algorithms are selected based on scenarios closely related to those in which they are to be applied. Defining outbreaks for the purposes of performance evaluation is also an area of considerable uncertainty. We used simulated outbreaks to describe and compare algorithm performance. Although we based disease transmission settings on epidemiological knowledge and attempted to test the algorithms over a variety of outbreak scenarios using a locally appropriate spatial structure, the evaluation may be not be an accurate representation of performance for actual hepatitis A outbreaks due to the simplification process involved or the simulation settings used.

Most algorithms currently used for automated surveillance are based on temporal monitoring methods [3]. The EARS algorithms have been found to be effective in a number of contexts for predicting and monitoring trends for influenza surveillance based on diagnostic and pre-diagnostic data [28-30]. Consideration of the epidemiology of the conditions under surveillance, public health priorities and response capacities are required to determine the relative importance of false alarm rate, sensitivity and timeliness for each particular surveillance application. In a routinely collected notifiable disease surveillance context where data transfer can be delayed, when one case does not automatically initiate public health action, and when the disease has a low reproductive number, small differences in the timeliness or sensitivity of algorithms may not significantly affect their usefulness. The need to monitor multiple data streams is also likely to influence the desired false alarm rate for surveillance. However, the cost of false alarms may not be high if they are linked with clear procedures that facilitate efficient epidemiological review of relevant data to determine if further investigation or heightened monitoring is warranted.

The increasingly timely availability of diagnostic health data provides opportunities for the application of automated surveillance methods to help ensure the early detection and control of outbreaks. We have described a general method for the surveillance of small area count data that only requires case data, although the model may be extended to include covariates. Postcode areas in Western Australia vary widely in terms of area and population size and are not ideal spatial units for surveillance. In Western Australia the size of postcode areas ranges from less than 0.7 square kilometres to over 700,000 square kilometres. However, the HMM may be applied to any available spatial areal unit, or applied to point-level data using distance-based models. The investigation of alternative methods to the deterministic nearest neighbours approach to identify clustered cases within the HMM, particularly given improved spatial data resolution, such as a Gaussian Spatial Exponential model [31], are likely to improve the model performance.

## Conclusion

The general Bayesian HMM performs well when compared with established limited-baseline temporal surveillance methods at low false alarm rates, and provides a viable addition to temporal surveillance algorithms in practice, particularly if high false alarm rates are problematic. We found that the use of different prior means for the HMM had only a small impact on model performance, suggesting that available historical data or surveillance goals should provide adequate basis for the specification of model parameters.

## Competing interests

The authors declare that they have no competing interests.

## Authors' contributions

REW and AJP designed the study, REW conducted the analysis and drafted the manuscript, and SE, GW and BV were involved in critically revising the manuscript. All surviving authors read and approved the final manuscript.

## Pre-publication history

The pre-publication history for this paper can be accessed here:

http://www.biomedcentral.com/1472-6947/9/39/prepub

## Supplementary Material

Additional file 1 **Supplementary material**. Contains additional results tables, and code for HMM, Early Aberration Reporting System (EARS) and negative binomial cusum (NBC) algorithms. Click here for file
