# Supplementary material for: Disease surveillance using a hidden Markov model
Source: BMC Med Inform Decis Mak. 2009 Aug 10;9:39. doi: 10.1186/1472-6947-9-39 (PMC2735038; doi:10.1186/1472-6947-9-39)
Supplement: Additional file 1 — Supplementary material. Contains additional results tables, and code for HMM, Early Aberration Reporting System (EARS) and negative binomial cusum (NBC) algorithms. [file 1472-6947-9-39-S1.pdf]

Supplement

Table 1. Algorithm performance statistics for smaller more clustered outbreaks (S2:n=62) for false alarm (FA) rates approximating 0.05 and 0.01.

Algorithm	mean FA rate	mean sensitivity	mean (median) timeliness	mean (median) adjusted timeliness
EARS C1	0.048	53.2	6.5 (0)	16.5 (23)
EARS C2	0.045	83.9	5.7 (1)	9.3 (4.5)
EARS C3	0.049	96.8	3.1 (0)	3.9 (0)
NBC 7	0.050	95.2	3.0 (1)	4.2 (1)
NBC 14	0.050	98.4	2.9 (1)	3.3 (1)
NBC 28	0.052	90.3	4.7 (1)	7.0 (1)
HMM 7	0.048	90.3	6.3 (3)	8.4 (4)
HMM 14	0.050	96.8	4.2 (2)	5.0 (2)
HMM 28	0.047	98.4	6.2 (4)	6.6 (4.5)
EARS C1	0.0004	11.3	8.1 (8)	25.8 (28)
EARS C2	0.008	11.3	6.7 (3)	25.6 (28)
EARS C3	0.008	25.8	5.3 (1.5)	22.1 (28)
NBC 7	0.008	58.1	8.3 (3.5)	16.5 (19)
NBC 14	0.010	75.8	5.7 (3)	11.1 (6.5)
NBC 28	0.012	75.8	6.9 (4)	12.0 (8)
HMM 7	0.010	79.0	8.1 (7)	12.2 (10.5)
HMM 14	0.010	96.8	6.0 (5)	6.7 (5)
HMM 28	0.010	98.4	6.5 (5)	6.9 (5)

Table 2. Algorithm performance statistics for larger less clustered outbreaks (S3:n=95) for false alarm (FA) rates approximating 0.05 and 0.01.

Algorithm	mean FA rate	mean sensitivity	mean (median) timeliness	mean (median) adjusted timeliness
EARS C1	0.049	55.8	5.8 (2)	15.6 (16)
EARS C2	0.048	93.7	4.9 (4)	6.4 (4)
EARS C3	0.050	94.7	3.1 (0.5)	4.4 (1)
NBC 7	0.050	97.9	2.8 (1)	3.3 (1)
NBC 14	0.050	98.9	2.5 (1)	2.8 (1)
NBC 28	0.055	97.9	3.6 (1)	4.2 (1)
HMM 7	0.049	98.9	3.3 (2)	3.6 (2)
HMM 14	0.050	97.9	4.6 (3)	5.1 (4)
HMM 28	0.047	95.8	5.6 (4)	6.6 (5)
EARS C1	0.0004	20.0	7.4 (8)	23.9 (28)
EARS C2	0.009	21.1	8.9 (5.5)	23.9 (28)
EARS C3	0.009	43.2	7.9 (5)	19.3 (28)
NBC 7	0.009	71.6	7.4 (6)	13.3 (11)
NBC 14	0.010	80.0	6.7 (6)	11.0 (8)
NBC 28	0.013	90.5	7.3 (7)	9.3 (7)
HMM 7	0.010	90.5	6.7 (6)	8.7 (7)
HMM 14	0.010	95.8	5.5 (5)	6.4 (5)
HMM 28	0.010	95.8	5.8 (5)	6.7 (5)

WinBUGS Hidden Markov model code

```
model

{

# model for initial time period t=1

	for (t in 1:1) {                                    # T time periods

	for (i in 1:N) {                                    # N areas

		x[1,i] <- y[1,i] + yn[1,i]            # monitor x = cases in area i + neighbours of i

		z[1,i] ~ dcat(pInit[1,i,1:K])         # hidden state z in one of K(=2) states

		pInit[1,i,1:K] ~ ddirch(alpha[1:K])        # initial probability of being in state z

		x[1,i] ~ dpois(mu[z[1,i]])                 # different poisson mean for each state

#cases in neighbouring areas: sum over neighbours for that time period

		yn[1,i] <- sum(Neigh[1,C[i]+1:C[i+1]])

	}

	}

# model for subsequent periods

	for (t in 2:T) {

	for (i in 1:N) {

		x[t,i] <- y[t,i] + yn[t,i]

		z[t,i] ~ dcat(p[z[t-1,i],1:K])

		x[t,i] ~ dpois(mu[z[t,i]])

# cases in neighbouring areas

		yn[t,i] <- sum(Neigh[t,C[i]+1:C[i+1]])

	}

	}

# neighbours

	for(t in 1:T) {

	for(i in 1:sumNumNeigh) {

		Neigh[t,i] <- y[t,adj[i]]
```

```
        }
        }
# priors on Poisson parameters of latent classes
        mu[1] ~ dgamma( 10,10)
        mu[2] ~ dgamma( 40,20) I(mu[1],)
# prior on transition matrix (gamma equivalent to Dirichlet)
    for(k in 1 : K) {
    for(l in 1 : K) {
                p[k,l] <- px[k,l]/sum(px[k,])
                px[k,l]   ~ dgamma(alpha[l],1)
        }
        }
# equilibrium allocation
        pi[1] <-  p[1,2]/(p[1,2]+p[2,1])
        pi[2] <- 1-pi[1]
# output for mapping or summary by day
        for (i in 1:N) {
                z7[i]<- z[7,i]
        }
}
```

R code for Early Aberration Reporting System (EARS) and negative binomial cusum (NBC) algorithms

```
# CDC EARS CUSUM C1 C2 C3

# calculate mean and sd for the last 7-days and the last 7-days offset by 2 days

        u71[i] = mean(x[(i-7):(i-1)])

        sd71[i] = sd(x[(i-7):(i-1)])

        u93[i] = mean(x[(i-9):(i-3)])

        sd93[i] = sd(x[(i-9):(i-3)])

# adjust any sd=0 to = 0.2 (to avoid dividing by zero)

        sd71[sd71==0]= 0.2

        sd93[sd93==0] = 0.2

# calculate cusums C1 (S1), C2 (S2), C3 (S3)

        S1[i] = max(0, (x[i] - (u71[i] + sd71[i]))/sd71[i])

        S2[i] = max(0, (x[i] - (u93[i] + sd93[i]))/sd93[i])

# for C3 past S2 values are only included in S3 if they are not >2

        S3a[i]=ifelse(S2[i] > thr, 0, S2[i])

        S3b[i] = sum(S2[i], S3a[(i-1):(i-2)])

        S3[i]=ifelse(x[i] <= u93[i], 0, S3b[i])
```

```
## Negative binomial cusum with baseline based on the last 7 days with a 2 day lag

# calculate mean and variance

        u93[i] = mean(x[(i-9):(i-3)])

        var93[i] = var(x[(i-9):(i-3)])

# adjustment if mean and or var = 0

        if(u93[i]==0) {u93[i]<-0.1}

        if(var93[i]==0) {var93[i]<-0.105*u93[i]}

# adjustment if mean < = var

        if(u93[i]>=var93[i]) {var93[i]<- u93[i]*1.05}

# calculate NB parameters c and r (the in control c)

        c93[i] = 1/((var93[i]/u93[i])-1)

        r93[i] = u93[i]*c93[i]

# calculate out of control mean value as 2sd > observed mean

        ux93[i] = u93[i] + 2*((var93[i])^0.5)

# calculate out of control cx value based on out of control mean and fixed r

        cx93[i] = r93[i]/ux93[i]

# calculate k based on c, cx, r

        firstlog9[i] = (c93[i]*(1+cx93[i]))/(cx93[i]*(1+c93[i]))

        secondlog9[i] = (1+c93[i])/(1+cx93[i])

        k93[i]=r93[i]*(log(firstlog9[i]))/(log(secondlog9[i]))

# calculate negative binomial statistic C93 (positive deviations)

        C93[i] <- max(0,C93[i-1] + x[i] - k93[i])
```
